# The Role of Emotion Dysregulation in Understanding Suicide Risk: A Systematic Review of the Literature

**DOI:** 10.3390/healthcare12020169

**Published:** 2024-01-10

**Authors:** Elena Rogante, Mariarosaria Cifrodelli, Salvatore Sarubbi, Alessandra Costanza, Denise Erbuto, Isabella Berardelli, Maurizio Pompili

**Affiliations:** 1 Department of Human Neurosciences, Sapienza University of Rome, Viale dell’Università 30, 00185 Rome, Italy; elena.rogante@uniroma1.it (E.R.); salvatore.sarubbi@uniroma1.it (S.S.); 2 Psychiatry Residency Training Program, Psychiatry Unit, Sant’Andrea Hospital, Sapienza University of Rome, Via di Grottarossa 1035, 00189 Rome, Italy; mariarosaria.cifrodelli@uniroma1.it; 3 Department of Psychiatry, Faculty of Medicine, University of Geneva (UNIGE), 1205 Geneva, Switzerland; alessandra.costanza@unige.ch; 4 Department of Neurosciences, Mental Health and Sensory Organs, Suicide Prevention Center, Sant’Andrea Hospital, Sapienza University of Rome, 00189 Rome, Italy; denise.erbuto@gmail.com (D.E.); isabella.berardelli@uniroma1.it (I.B.)

**Keywords:** difficulties in emotional regulation, suicidal ideation, suicide attempt, suicide risk, systematic review

## Abstract

Suicide prevention represents a global imperative, and efforts to identify potential risk factors are intensifying. Among these, emotional regulation abilities represent a transdiagnostic component that may have an impactful influence on suicidal ideation and behavior. Therefore, the present systematic review aimed to investigate the association between emotion dysregulation and suicidal ideation and/or behavior in adult participants. The review followed PRISMA guidelines, and the research was performed through four major electronic databases (PubMed/MEDLINE, Scopus, PsycInfo, and Web of Science) for relevant titles/abstracts published from January 2013 to September 2023. The review included original studies published in peer-reviewed journals and in English that assessed the relationship between emotional regulation, as measured by the Difficulties in Emotional Regulation Scale (DERS), and suicidal ideation and/or behavior. In total, 44 studies were considered eligible, and the results mostly revealed significant positive associations between emotion dysregulation and suicidal ideation, while the findings on suicide attempts were more inconsistent. Furthermore, the findings also confirmed the role of emotion dysregulation as a mediator between suicide and other variables. Given these results, it is important to continue investigating these constructs and conduct accurate assessments to implement effective person-centered interventions.

## 1. Introduction

Suicide is a significant public health concern, with more than 700,000 people dying by suicide every year [1]. Consequently, research aimed at building a deeper understanding of suicide and identifying risk factors and their interactions is key to implementing effective suicide prevention strategies. Among the psychological factors involved in suicide risk in the general population and patients with psychiatric disorders, emotion dysregulation seems to be a prominent feature highlighted by many related studies. Gratz and Roemer [2] defined emotional regulation as a multidimensional construct resulting from the combination of different abilities: awareness and understanding of emotions, acceptance of emotions, ability to control impulses, ability to pursue specific goals even when experiencing negative affect, ability to use emotional regulation strategies with flexibility and appropriateness for situations, and ability to modulate emotional responses to meet individuals’ aims and demands. An individual experiences difficulties in emotional regulation when one or more of these skills are absent [2], as measured by the Difficulties in Emotional Regulation Scale (DERS) [2].

Hence, the ways people regulate their emotions and emotional distress are different, and such differences are transdiagnostic risk factors for psychopathological mechanisms. They are involved in suicide risk through various pathways [3,4]. Accordingly, several psychological theories have focused on escaping emotional or psychological pain as a possible role in suicide. Shneidman [5] proposed that suicide is caused by unbearable and intolerable psychological pain, named ‘*psychache*’. Psychache combined with hopelessness is essential for the development of suicidal ideation, which becomes stronger in the presence of disrupted connectedness and emotional dysregulation. The interpersonal theory of suicide (IPTS) [6] states that people develop suicidal ideation because of unmet interpersonal needs, manifested as elevated perceived burdensomeness and thwarted belongingness [6]. In this theoretical framework, emotion dysregulation has been consistently linked with interpersonal difficulties [7,8]; specifically, people with difficulty regulating their emotions could develop interpersonal rejection and experience feelings of social isolation and the perception of being a burden during stressful periods as a consequence of increased suicidal ideation [9]. Moreover, emotionally dysregulated individuals may also exhibit impulsive and dysregulated behaviors that increase their capability for suicide and increase their likelihood of attempting suicide in the presence of ideation [10,11]. The Integrated Motivational–Volitional Model of Suicidal Behavior Model [12] (IMV) expands on the IPTS and has three phases: premotivational with diathesis and triggering events; motivational, which leads to the development of suicidal ideation; and volitional, which supports the enactment of suicidal behavior. These phases are influenced by moderators that can intensify or reduce the likelihood of developing suicidal ideation or engaging in suicidal behavior. Among these moderators, feelings of entrapment triggered by perceptions of defeat and/or humiliation are central to the motivational phase. These feelings, consequent to negative self-appraisals, support the belief that suicide is the only solution and originate from a dysfunctional affect regulation strategy combined with social isolation factors. The three-step theory of suicide [13] highlights the role of pain, both emotional and psychological, in comprehending suicide risk. The theory links the copresence of pain and hopelessness to the development of suicidal ideation, which is amplified by disrupted social connectedness. Within this model, the transition from ideation to suicide behavior is directed by the capability for suicide, which depends on genetic, environmental, and social factors. In this context, it is also essential to consider the emerging role of epigenetics, which has been recurrently associated with suicide risk and emotional regulation [14]. Therefore, perceiving oneself as a burden, being disconnected from the social environment, and experiencing a high degree of psychological pain are associated with unsuccessful emotional coping and are elements implicated in the theories mentioned above [5,6,12,13]. Accordingly, studies have shown that emotion dysregulation eases the development of suicidal ideation and behavior [15,16,17,18]. For instance, de la Torre-Luque and colleagues [19] examined different trajectories characterized by earlier emotional dysregulation associated with a higher risk of lifetime suicide attempts, as well as other proximal factors.

Disrupted regulation of emotions may be one of the factors that explains why psychological pain can lead to suicidal ideation and behavior. According to the review conducted by Colmenero-Navarrete et al. [20], crucial variables of suicide models, such as perceived burdensomeness, hopelessness, psychache, and entrapment, are associated with negative emotional responses and may be involved in the process that leads to the development of suicidal ideation and/or behavior.

Previous studies and systematic reviews have already examined the associations between suicide and specific emotional regulation difficulties and maladaptive strategies [21,22,23,24,25,26] in adult and adolescent populations. In particular, Anestis et al. [21] examined a sample of undergraduate students, and the results indicated that those with difficulties in emotional regulation strategies exhibited higher levels of suicidal ideation. Pisani et al. [22] reported that emotion dysregulation was associated with increased risk for previous suicide attempts, above and beyond the effects of depressive symptoms and demographic factors. On the other hand, the review by Turton et al. [24] showed positive associations between emotion dysregulation and suicidal ideation and attempts. Still, this association was not significant when controlling for other psychological variables. Nevertheless, constant updates are necessary to maintain interest and knowledge on this crucial topic.

Considering the growing interest in suicide prevention and based on the above-mentioned theoretical frameworks that consider the importance of emotion dysregulation in suicide risk, the primary aim of this systematic review was to evaluate empirical findings that report on the relationship between emotion dysregulation and suicide risk (suicidal ideation and/or attempts) in the adult population to enrich the literature and integrate new and future lines of research.

## 2. Materials and Methods

A systematic literature review was performed according to the Preferred Reporting Items for Systematic Reviews and Meta-Analyses (PRISMA) guidelines [27].

### 2.1. Search Strategy

**Figure 1:**
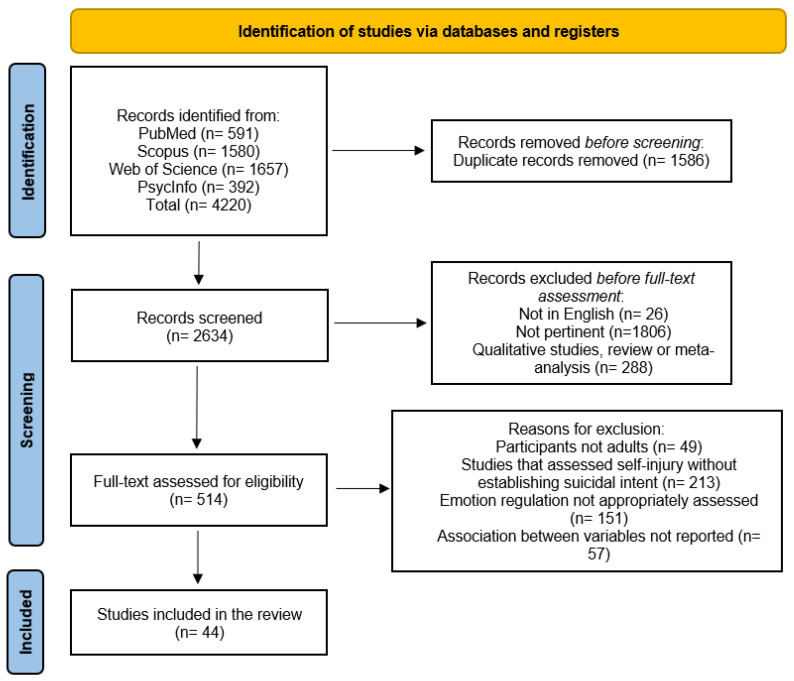
PRISMA flow chart.

To be inclusive, we systematically searched 4 major electronic databases of medical and social science research papers (PubMed/MEDLINE, Scopus, PsycINFO, and Web of Science) for relevant titles and abstracts published between January 2013 and September 2023. The following terms were combined to search the databases in titles/abstracts (TAs): “Emotion* regulation” OR “Emotion* dysregulation” OR “Affect regulation” OR “Affect dysregulation” AND “Suicide*”. Filters were used to limit the results to the English language. The search identified 4220 papers. See Figure 1 for details of the study selection process.

### 2.2. Eligibility Criteria

This review included original articles that explicitly discussed the association between difficulties in emotional regulation and suicidal ideation and behavior in the adult population. When a title or abstract indicated an eligible study, the full-text article was obtained and carefully examined to assess its relevance for the review’s aims. Articles needed to meet the following eligibility criteria: (a) include a measure of suicidal ideation or behavior and (b) include the Difficulties in Emotional Regulation Scale (DERS) [2] as a measure of emotional regulation. The Difficulties in Emotional Regulation Scale (DERS) [2] is one of the most common measures used to assess emotion dysregulation. It consists of 36 items and provides a total score of difficulties in regulating emotions and 6 subscale scores that reflect the dimensions considered by the authors (emotional clarity [Clarity], emotional awareness (Awareness), accepting one’s emotional experiences (Nonacceptance), controlling impulsive behavior (Impulse), engaging in goal-directed behavior (Goals), and accessing emotional regulation strategies while distressed (Strategies)) [2]. Because of variations in the definition of emotional regulation, papers needed to have utilized this measure to ensure the consistency of definitions across all papers. In addition, (c) participants needed to be adults (aged 18 years and older) and be (d) quantitative research papers with a case-control, cohort, or cross-sectional design. The exclusion criteria were as follows: (a) published before 2013; (b) assessed self-injury without establishing suicidal intent; (c) did not use DERS to assess difficulties in emotional regulation (the results included DERS total scores and/or scores for each dimension); (d) did not investigate the association between suicidal ideation and/or behavior and emotion dysregulation; (d) not published in peer-reviewed journals; (d) not published in English; and if they were (d) qualitative research, case studies, meta-analytical reviews, systematic reviews, narrative reviews, or book chapters.

### 2.3. Study Selection and Data Collection

The authors independently extracted and reviewed the studies using a two-step process: (1) screening and selecting based on the article’s title and abstract and (2) screening and selecting based on the full text. A data extraction spreadsheet was developed, adding the author(s), publication year, country, sample characteristics (population type and sample size), study design, main suicide outcomes and respective measures, and main results. Discussions among the senior authors, who also independently read all the articles, resolved potential disagreements regarding article inclusion and data collection.

### 2.4. Study Inclusion

The PRISMA flowchart of the study selection process is presented in Figure 1. Electronic searches identified 4220 publications. After excluding duplicates (n = 1586), 2634 abstracts and titles were screened for suitability. After non-English studies (n = 26); nonpertinent studies (n = 1806); and qualitative studies, reviews, and meta-analyses were removed (n = 288), 514 full-text titles were assessed for eligibility, with 470 records excluded because they were not in line with the aims and inclusion criteria of our review (see Figure 1 for detailed reasons). Any disagreements regarding study eligibility were resolved following consensus discussions among three authors (S.S., M.C. and E.R.). Overall, 44 studies were included in the present review.

### 2.5. Study Quality Assessment

The following criteria, adapted from the Study Quality Assessment Tools edited by the National Institute of Health (NIH), were used to assess the quality of the articles that evaluated the association between suicidal ideation, and/or behavior and difficulties in emotional regulation: (1) research question or objective/s clearly stated; (2) representativeness of the sample/absence of selection bias (0.5 point) and presence of clearly stated inclusion and exclusion criteria (0.5 point); (3) presence of a control sample; (4) justification of sample size or presence of a power analysis; (5) presence of more than one assessment point (i.e., baseline and follow-up); (6) presence of evidence-based measures to assess the outcome (0.5 point for self-report; 1 point for clinician report, as clinician-reported information is generally considered more reliable); and (7) presence of covariates adjusted statistically for their impact on the outcome. A score between 0 and 1 was assigned to each criterion, with an overall quality score ranging from 0 to 7. The coauthors first examined the selected manuscripts separately; second, they discussed the scores together. Consensus with the senior researcher (I.B.) resolved any discrepancies or disagreements. After assigning a score to each study, the authors divided the studies into 3 categories: low-quality studies (0–2 points), moderate-quality studies (3–5 points), and good-quality studies (6–7 points). The findings from studies with a low-quality score were interpreted with particular caution. The mean score of all of the included studies was 3.47.

## 3. Results

The systematic search identified 44 original research articles [11,28,29,30,31,32,33,34,35,36,37,38,39,40,41,42,43,44,45,46,47,48,49,50,51,52,53,54,55,56,57,58,59,60,61,62,63,64,65,66,67,68,69,70] that assessed the relationship between emotional regulation and suicidal ideation or suicide attempts in clinical and nonclinical samples. The study characteristics are summarized in Table 1.

### 3.1. Study Characteristics

The publications included in the review were published in the USA (n = 26; 59.1%), Italy (n = 5; 11.4%), Iran (n = 3; 6.8%), Canada (n = 3; 6.8%), China (n = 2; 4.5%), Spain (n = 2; 4.5%), Scotland (n = 1; 2.3%), the UK (n = 1; 2.3%), and Korea (n = 1; 2.3%). The sample sizes ranged from 60 to 2693 participants. Eighteen studies recruited clinical samples predominantly from mental health inpatient units [30,34,35,39,44,45,51,56,60,61,67]. Some studies referred to specific diagnoses, such as substance-use disorder [11,35,48], bipolar disorder [51,52,53], and eating disorder [34,39,54]. Five studies recruited mixed samples of both clinical and nonclinical participants [11,38,49,52,55]. Twenty-one studies assessed nonclinical samples; eleven studies involved undergraduate students [29,32,40,47,58,59,64,65,68,69], two studies involved community participants [28,70], and two involved prisoners [37,66]. Moreover, Decker et al. [33] recruited veterans; Khazem and Anestis [42] recruited participants online; Mata-Greve et al. [46] enrolled LGBTQ individuals, as well as individuals who identified as Black, Indigenous, and People of Color; Orr et al. [50] recruited opioid-using adults with chronic pain; and Rodriguez-Cano et al. [57] involved daily smokers. Lemaigre and Taylor [43] recruited participants from a community group with past or current suicidal ideation and behavior. All of the studies used a cross-sectional design, except for those of Al-Dajani et al. [28], Mata-Greve et al. [46], Miranda et al. [47], and Raudales et al. [56], who used longitudinal data.

### 3.2. Suicidal Ideation and Suicidal Behavior Measures

The studies included in the review were particularly heterogeneous for both the suicidal outcomes explored and the measures used. Specifically, 12 studies [28,31,36,50,51,52,53,55,57,64,68,69] focused on suicidal ideation only, 8 studies [11,29,35,39,41,46,54,60] considered suicide behavior as the main outcome, and the remaining 24 studies included both constructs in their analyses. The Beck Scale for Suicide Ideation (BSS) [71] was the most common measure used in the assessment; it was used in 11 studies [28,45,47,55,58,59,63,65,68,69,70], followed by the Suicidal Behavior Questionnaire—Revised (SBQ-R) [72], which was used in 6 studies [28,29,37,40,43,46], and the Scale of Suicide Ideation (SSI) [73], which was also used in 6 studies [30,38,48,51,52,53]. The Columbia-Suicide Severity Rating Scale (C-SSRS) [74] was used in 4 studies [30,33,56,60], while 5 studies [42,54,59,65,66] used questions adapted from the Self-Injurious Thoughts and Behaviors Interview (SITBI) [75]. Item 9 of the Beck Depression Inventory (BDI) [76] was used to assess suicidal ideation in three different studies [32,50,57]. In contrast, direct questions, mainly used to investigate the presence of suicide attempts, were used in nine studies [32,38,39,41,44,47,48,61,67].

### 3.3. Emotional Regulation and Suicidal Ideation

#### 3.3.1. Emotional Regulation and Suicidal Ideation in Clinical Samples

Fourteen studies assessed the association between emotional dysregulation and suicidal ideation in clinical samples. Overall, DER were mostly positively related to suicidal ideation. Suicidal ideation was significantly and positively correlated with the DERS total score in three samples of adults with suicidal ideation [31,55,63], in males with substance-use disorders [48], and in three studies on patients with bipolar disorder [51,52,53]. The same association was observed in a longitudinal study of inpatients with PTSD [56], in which emotional dysregulation assessed at the 3-week follow-up was associated with suicidal ideation at the 6-week follow-up, and in a sample of psychiatric inpatients [61], in which the DERS total score was related to both passive and active suicidal ideation and to its intensity. A study by Martin and colleagues [45] showed a positive correlation between the DERS total score and suicidal ideation severity. The only study in which emotional dysregulation was not associated with suicide risk severity was the one by Denning and colleagues [34] on a sample of patients with eating disorders. Finally, Mallorqui-Baguè et al. [44] compared patients with gambling disorder with and without suicidal ideation and reported that the DERS total score was greater in the first group and that emotional dysregulation and gambling disorder severity increased the risk of suicide through worsening of psychopathological conditions.

#### 3.3.2. Specific Dimensions of Emotional Regulation

Related to the associations between suicidal ideation and specific dimensions of the DERS assessed in five studies, Baer and colleagues [30] found an association with the flexible use of emotion regulation strategies in inpatients hospitalized for suicide risk. Ghorbani et al. [38] showed that all the dimensions were positively associated with suicidal ideation and that impulse control difficulties and reduced goal-directed behavior were significant predictors of suicide risk in treatment-seeking alcohol-dependent outpatients. Mewhile Ponzoni et al. [55] found an association with all of the dimensions of DERS, except difficulties with emotional awareness. Furthermore, in the study by Palagini et al. [53], significant associations were detected, with difficulties controlling impulsivity, difficulties with emotional awareness, and flexible use of emotional regulation strategies; these dimensions were found to be significant predictors of passive and active suicidal ideation and suicide plans, while impulse control difficulties predicted only passive suicidal ideation. Lastly, in Turton et al. [63], only impulse control difficulties, reduced goal-directed behavior, and flexible use of emotional regulation strategies significantly related to suicidal ideation.

#### 3.3.3. Emotional Regulation and Suicidal Ideation in Nonclinical Samples

Emotional dysregulation was positively related to suicidal ideation in most of the 18 studies with nonclinical samples. Suicidal ideation was significantly associated with the DERS total score in eight studies conducted on university students, as highlighted by Zemestani et al. [69], Zeifman et al. [68], and Rogers and Joiner [58]. According to Clapham and colleagues [32], emotional dysregulation accounted for 36.7% of the variance in suicidal ideation. In contrast, after controlling for depression and sex, only the dimension of flexible use of emotional regulation strategies remained significantly associated with suicidal ideation. Duggan et al. [36], who considered suicide-related rumination due to body image experiences, reported a significant association with difficulties in emotional regulation. The study by Haliczer and colleagues [40], again highlighted the positive association between DERS scores and suicidal ideation. The authors revealed an interaction effect of emotional dysregulation and self-damaging behavior on suicide risk, with engagement in more forms of such behaviors (NSSI, eating disorders, etc.) conferring a higher risk for suicide, particularly in the context of greater emotional dysregulation. Moreover, greater emotional dysregulation had an indirect effect on elevated suicide risk via several self-damaging behaviors. In the study by Miranda et al. [47], which administered measures across different time points, there was a weak but positive correlation between baseline DERS scores and suicidal ideation two years later; however, in the study by Van Eck and colleagues [64], students who reported suicidal ideation had significantly higher scores on emotional dysregulation constructs. Weak but significant positive correlations between DERS and both passive and active suicidal ideation were found by Decker et al. [33] in U.S. veterans and by Fadoir et al. [37] in a sample of offenders. Positive associations were also found in adults recruited online [43], in community participants [70], in opioid-using adults with chronic pain [50], and in daily smokers [57], where there was an interaction between emotional dysregulation and hazardous drinking status in determining suicidal ideation. In a longitudinal study by Al-Dajani et al. [28], emotional dysregulation was significantly correlated with suicidal ideation both at baseline and at follow-up, with emotional dysregulation at baseline predicting suicidal ideation at follow-up. According to Khazem and Anestis [42], adults with suicidal ideation reported higher scores in emotional dysregulation than adults without suicidal ideation. Moreover, Neacsiu and colleagues [49] reported that the DERS score was a significant predictor of suicidal ideation, accounting for 11% of the variance. Finally, a study of male prisoners with antisocial personality disorder (ASPD) or borderline personality disorder (BPD) [66] presented different results: In the sample with ASPD, all the dimensions of DERS showed positive and significant correlations with lifetime and last-year suicidal ideation, while in the sample with BPD, all the dimensions of the DERS showed positive and significant correlations with last-year suicidal ideation, but only impulse control difficulties, reduced goal-directed behavior, and flexible use of emotional regulation strategies were related to lifetime suicidal ideation.

### 3.4. Emotional Regulation and Suicide Attempts

#### 3.4.1. Emotional Regulation and Suicide Attempts in Clinical Samples

The association between suicide attempts and emotional regulation was assessed in 15 studies recruiting clinical participants. In contrast with the findings of studies on suicidal ideation, the results of these studies were mixed. In Anestis et al. [11], a sample of adult inpatients with substance-use disorders showed significant and positive correlations between suicide attempts and DERS scores. These associations remained significant when NSSI was entered as a mediator into the model. In contrast, another sample of adults with substance-use disorders [35] revealed no significant relationship between DERS score and history of suicide attempts. Rufino et al. [61] reported that emotional dysregulation and its interaction, at moderate and high levels, with nightmare frequency were significantly related to previous suicide attempts, as was the interaction between nightmares and all DERS subscales, except difficulties in engaging in goal-directed behaviors. In the study by Martin et al. [45] on inpatients with psychiatric disorders, there was no significant correlation between suicide attempts or suicidality during hospitalization and emotional dysregulation. Denning et al. [34] reported that, at a bivariate level, all the dimensions assessed by the DERS, except difficulties with emotional awareness, were related to lifetime suicide attempts. In contrast, limited access to adaptive emotional regulation strategies, difficulties engaging in goal-oriented behaviors, and engaging in impulsive behavior when experiencing negative emotions were found to be predictors of suicide attempt frequency even after entering depressive symptoms into the model. Similarly to the mean comparisons, Ghorbani et al. [38] reported higher DERS scores in patients with lifetime suicide attempts, as also highlighted in a sample of patients with eating disorders recruited by Gomez-Exposito and colleagues [39]. Baer et al. [30] reported that only the dimension of flexible use of emotional regulation strategies was significantly greater in individuals with multiple versus single suicide attempts, and according to multiple logistic regression, none of the DERS dimensions differentiated between individuals with single versus multiple suicide attempts. According to Harris and colleagues [41], significantly higher total DERS scores were found for patients with previous suicide attempts. Still, emotional dysregulation was not independently associated with a history of suicide attempt. In patients with gambling disorder and suicidal ideation [44], there were no significant differences in difficulties in emotional regulation between those with suicide attempts and those without, while in a sample of patients with major depressive disorder [49], those with a history of suicide attempts had higher DERS scores than did those without. In contrast, Yoon et al. [67] found no difference in emotional dysregulation between suicide ideators and attempters, nor did Silvers et al. [62] in a sample of patients with borderline personality disorder with and without suicide attempts. Finally, in a sample of patients with suicidal ideation in the past year [63], individuals with multiple suicide attempts reported greater emotional dysregulation than did those without a history of suicide attempts, but there was no difference in DERS score between single and multiple attempters or between single and nonattempters; however, in the study by Pisetsky et al. [54], the authors did not detect any differences between individuals with or without suicide attempts.

#### 3.4.2. Emotional Regulation and Suicide Attempts in Nonclinical Samples

The association between suicidal behavior and emotional regulation was assessed in 12 studies recruiting nonclinical samples. As in studies with clinical participants, the results were conflicting. Specifically, in university students, there were positive and significant correlations between suicide attempts and emotional dysregulation in both Anestis et al. [11], Miranda et al. [47], and Rogers and Joiner [58]; however, the latter also suggested that the DERS score was unrelated to a lifetime of suicide attempts when included in logistic regression. Linear regression with emotional dysregulation as the independent variable and suicide attempt as the dependent variable was also not significant [32]. Compared with the mean comparisons, university students with previous suicide attempts had significantly higher DERS scores [29]. Furthermore, the DERS mean score differed significantly among students with multiple attempts, one attempt, and no attempt [47], and among those who reported aborted, interrupted, or actual attempts, with actual attempts showing higher mean scores [59]. Finally, in Yang et al. [65], both suicide ideators and suicide attempters scored significantly higher on DERS than nonideators, and suicide attempters scored significantly higher on emotional dysregulation than suicide ideators; moreover, according to the results of the multinomial regression model, the dimensions of reduced goal-directed behavior, difficulties with emotional clarity, and flexible use of emotional regulation strategies could significantly discriminate suicide ideators from nonideators; additionally, impulse-control difficulties could significantly discriminate suicide attempters from nonideators, and difficulties with emotional awareness, difficulties with emotional clarity, and impulse control difficulties could significantly discriminate suicide attempters from suicide ideators. Weak, but significant positive correlations between DERS scores and suicide attempts were found in a sample of veterans [33], and emotional dysregulation remained a significant predictor of suicide attempts when controlling for other possible confounders, except depression and PTSD. In a sample of adults recruited online, Khazem and Anestis [42] showed that individuals with previous suicide attempts had higher scores regarding difficulties in emotional regulation than those without suicide attempts. In a longitudinal study by Mata-Greve and colleagues [46], emotional dysregulation measured at T2 was positively related to suicide behavior at T3. In contrast, the total indirect effect of cultural stressors of suicide at T1 on suicide behavior at T3 via DERS at T2 was not significant. Finally, Yang et al. [66] showed that, in prisoners with antisocial personality disorder, there were positive and significant correlations between all DERS dimensions and the last year’s suicide plan, while only impulse control difficulties and flexible use of emotional regulation strategies were correlated with the previous year’s suicide attempts, whereas in prisoners with borderline personality disorder, nonacceptance of emotional responses, flexible use of emotional regulation strategies, and impulse control difficulties were related to the previous year’s suicide plans, and neither of the subscales of DERS showed a significant correlation with the previous year’s suicide attempts.

### 3.5. Emotional Regulation as a Mediator

Fifteen of the eligible papers included results on emotional dysregulation as a mediator between suicidal ideation or attempts and other variables. Difficulties in emotional regulation were found to mediate the relationships between suicidal ideation and NSSI [36,70], childhood trauma [43], psychopathy [37], cannabis-use problems [50], perfectionistic concerns and strivings [68], disrythmicity of social aspects [52,53], depressive symptoms [53], vulnerable narcissism [55], posttraumatic stress disorder [56], avoidant and anxious attachment [63], and sleep disturbances [69]. Moreover, specific dimensions of emotional dysregulation were tested as mediators between suicidal ideation and other variables; for example, in Mohammadzadeh et al. [48], difficulties with emotional awareness, nonacceptance of emotional responses, and flexible use of emotional regulation strategies mediated the association between childhood trauma and suicidal ideation, while Raudales et al. [56] showed that the flexible use of emotional regulation strategies, impulse control difficulties, reduced goal-directed behavior, and difficulties with emotional clarity mediated the association between PTSD and suicidal ideation. According to Van Eck et al. [64], emotional regulation deficits resulting from accepting negative emotions, emotional awareness, and goal-oriented behavior moderate the indirect effect of ADHD on suicidal ideation. Finally, emotional dysregulation acts as a mediator in the association between anger and suicide attempt [29], while the indirect effect of PTSD on suicide attempt through difficulties in emotional regulation is not significant [56].

## 4. Discussion

The present systematic review identified 44 relevant studies that investigated the relationship between emotional dysregulation, as assessed by the Difficulties in Emotional Regulation Scale (DERS) [2], and suicide in adults from both the clinical and general population. The focus was on disrupted emotional regulation processes such as difficulties with emotional clarity and awareness, difficulties in accepting one’s emotional experiences, controlling impulsive behavior, engaging in goal-directed behavior, and accessing flexible emotional regulation strategies.

Overall, the results confirmed significant associations between emotional dysregulation and suicidal ideation, while the findings related to suicidal behavior were more conflicting. Specifically, suicidal ideation showed weak to moderate associations with the global score of difficulties in emotional regulation and with specific dimensions of this construct in both clinical and nonclinical populations, except for one study on patients with eating disorders [34]. These findings may indicate that the perceived inability to overcome emotional demands may represent a salient factor that leads to suicidal ideation and it is seen as an alternative strategy for managing intolerable emotions [77,78]. On the other hand, the inclusion of other variables, such as psychopathological aspects, to explain suicidal ideation decreased or eliminated the contribution of emotional dysregulation. For example, a study by Clapham and colleagues [32] showed that the predictive value of emotional dysregulation on suicidal ideation becomes nonsignificant when introducing depressive symptoms and gender in regression. This result could reflect a more marginal role of emotional regulation strategies, which may indirectly influence suicide risk through other factors (i.e., psychiatric diagnoses).

As already argued, findings related to the association between difficulties in emotional regulation and suicide attempts were more inconsistent. Several studies did not find statistically significant associations [30,32,35,45] or evidence of emotional dysregulation between patients with and without suicide attempts [44,54,62,63,67]. These results are consistent with those of previous studies [21,78]. These findings may be explained by the fact that limited access to adaptive emotional regulation processes is not directly linked to suicide attempt but rather amplified through a dynamic association with suicidal ideation [20]. Moreover, in some of those studies [41,58] in which a significant association was detected, the introduction of other variables overcame the role of emotional dysregulation, suggesting that suicide attempt may be dependent on other aspects. For example, borderline personality disorder or posttraumatic stress disorder, which are strongly characterized by disrupted emotions, may represent more powerful predictors of suicide attempt [24].

In general, emotional dysregulation was found to mediate the relationship between suicidal ideation or attempt and various variables (i.e., childhood trauma, avoidant and anxious attachment, sleep disturbances, depressive symptoms, NSSI, etc.), except in one study [56]. These findings can be explained by considering difficulties in emotional regulation as a crucial factor that connects psychological aspects and suicide risk; consequently, emotional regulation may act as a mediator throughout the entire process, from suicidal ideation to suicide attempts [24]. From this perspective, it would be helpful to examine the association between emotional dysregulation and the constructs included in the principal theories on suicide, such as psychache, perceived burdensomeness, lack of belongin, and hopelessness, to provide a comprehensive framework in which suicide risk may be more intelligible. Furthermore, it is important to consider and expand the recent focus on the epigenetic modulation of genes involved in stress-response systems. This finding suggests the crucial role of early experiences in shaping the development of adaptive emotional, behavioral, and cognitive phenotypes [79]. Specifically, it has been argued that HPA axis dysfunction and DNA methylation subsequent to genetic and familial predisposition and to the presence of early life adversities may be responsible for stable modifications in the expression of genes associated with emotional regulation strategies [80]. According to Turecki and colleagues [81], individuals with early adversities, including both internalizing and externalizing behaviors, altered brain structure, and impaired executive function, all of which are traits that overlap with the suicidal phenotype, have a greater risk of developing pathological traits and emotional dysregulation. Finally, the role of the autonomic nervous system should not be omitted, as conceptualized by Porges [82] in the Polyvagal theory. It represents a functional map of the development of behavioral, emotional, and physiological reactivity to threats or positive experiences. This framework provides a neurophysiological substrate for processing feelings by higher brain structures. When the individual perceives negative states, this condition may evolve into feelings of anxiety or specific emotions such as fear or anger.

On the contrary, when the perceived feelings are positive and are associated with tranquillity, access to interpersonal availability and co-regulation, and experiencing feelings of trust, love, and intimacy [83]. Therefore, the nervous system seems to determine the range of emotional expression and the ability to regulate affective states, providing a plausible explanation of several social, emotional, and communication behaviors and disorders [84]. In light of these findings, the relationship between difficulties in emotional regulation and suicide risk is multifaceted and interconnected, and would benefit from increased interest in research that clarifies the role of disrupted emotional strategies with more sophisticated designs and analyses that allow us to determine pathways and infer causal bonds. To achieve these goals, overcoming some specific shortcomings and improving statistical methodology to investigate the predictive role of emotional regulation and enhance the representativeness of the results would be helpful. Reaching comprehensive knowledge of the pathways that lead to suicide risk is the key to implementing effective prevention strategies.

### Limitations

This systematic review needs to be interpreted in light of several limitations. First, the quality assessment of the studies included was relatively low, indicating a weak to moderate quality. Specifically, the assessment revealed the presence of selection bias in several studies, indicating poor representativeness of the population; for example, studies in which university students were recruited should be generalized with caution as this type of sample may not reflect the characteristics of the general population. The conclusions of studies recruiting individuals with specific diagnoses may not apply to other psychopathological conditions. From our perspective, the heterogeneity of subpopulations may restrict the application of our results to the general population. However, the presence of both clinical and non-clinical samples represents a critical attempt to be as representative as possible. Second, only four studies used a longitudinal design to investigate the target variables, preventing the possibility of making assumptions about the direction of the effects highlighted by the results.

Moreover, most of the studies used self-report measures to assess emotional dysregulation and suicide risk, exposing the data to recall bias and social desirability. The heterogeneity of the measures used to assess suicidal ideation and behavior represented another weakness for comparing findings across studies, which prevented a fair comparison and generalization of the results and narrowed the opportunity to draw solid conclusions. The significance of the present study was further limited by the lack of a meta-analytical design, which was not considered appropriate due to the variety of the samples included and the different methodological approaches. It is also important to explain the choice to exclude studies involving individuals younger than 18. This decision was sustained by the instability of emotional regulation and the numerous neurodevelopmental and social changes that intervene during adolescence [85]. Finally, the authors included only the studies that used DERS to assess emotional regulation. However, there was a wide range of conceptualizations and measures of emotional regulation available to minimize the heterogeneity of the related studies, and they adopted a consistent definition of the construct that reflected an overall process rather than specific strategies for controlling and expressing thoughts and emotions.

## 5. Conclusions

Overall, from the findings highlighted within the review, an association between emotional dysregulation and suicidal ideation and behavior emerged; however, the results were sometimes conflicting, and the studies presented some weaknesses. As the ability to regulate emotions has been proven to be a transdiagnostic element that has a modest impact on psychopathology, it is important to investigate this construct. It would be helpful to investigate the variables longitudinally to establish the predictive value of emotional regulation and the causal link. Furthermore, it may be useful to adopt more complex statistical approaches that assist with determining pathways, from emotional regulation to suicide risk, and to analyze the role of other variables both as confounders (i.e., sex and psychiatric diagnosis) and as mediators (i.e., hopelessness and psychache) to provide a comprehensive framework. Finally, it is recommended that more representative samples be recruited to amplify the generalizability of the conclusions.

From a preventative perspective, the complex interactions that emerged from the review underline the need to conduct a thorough and personalized assessment to identify areas of vulnerability and explore the impact on mental well-being. Moreover, interventions that endorse the knowledge and management of emotions should be considered. For example, dialectical behavioral therapy aims to improve skills related to emotional regulation through practice and increasing awareness [86,87].

## Figures and Tables

**Table 1:** Study characteristics and findings.

Authors and Year	Country	Sample Characteristics	Design	Main Suicidal Outcomes and Measures Used	Main Findings	Quality Score
Al-Dajani et al., 2019 [28]	Canada	101 community participants recruited through advertisement	Longitudinal	SI through18 items of the BSS and 2 items of the SBQ-14	ED is significantly and positively correlated with SI at baseline and at follow-up. Baseline ED predicted SI at follow-up. Finally, the belief that suicide is a solution to a problem did not moderate the ED-SI longitudinal relationship.	4
Ammerman et al., 2014 [29]	USA	2295 undergraduate students	Cross-sectional	SA through 1 item of the SBQ-R	Those with previous SAs had significantly higher DERS scores. DERS was a mediator in the relationship between anger and SA.	3
Anestis et al., 2014 [11]	USA	Study 1: 1317 undergraduate students; Study 2: 93 adult inpatients in treatment for SUD	Cross-sectional	Study 1: SA through MEPOS Study 2: SA through L-SASI	The results showed significant and positive correlations between SAs and DERS scores. DERS directly affected SA, which remained significant when NSSI was entered into the model.	3
Baer et al., 2018 [30]	USA	186 psychiatric inpatients hospitalized for SI or SA and with at least one SA	Cross-sectional	SI through SSI and C-SSRS SA through C-SSRS	Worst lifetime SI was significantly and positively correlated with the “Strategies” dimension of DERS. The results of multiple linear regression with SI as a dependent variable were not significant. Individuals with multiple SAs scored higher on the Strategies subscale than individuals with a single attempt. In multiple logistic regression, no DERS subscale significantly differentiated between individuals with single vs. multiple SA.	4
Bentley et al., 2018 [31]	USA	150 inpatients with SI and SA	Cross-sectional	SI through item 9 of PHQ-9	There was a positive and significant correlation between SI and DERS scores.	3
Clapham et al., 2022 [32]	USA	708 undergraduate students	Cross-sectional	SI through item 9 of BDI SA through 1 dichotomic question	The linear regression with ED as an independent variable and SI as a dependent variable was significant, accounting for 36.7% of the variance. After controlling for depression and gender, the “Strategies” dimension of DERS was the only subscale significantly associated with SI. The linear regression with ED as an independent variable and SA as a dependent variable was not significant.	3
Decker et al., 2019 [33]	USA	278 U.S. veterans	Cross-sectional	SI and SA through C-SSRS	There were weak significant positive correlations between DERS and passive SI, active SI, and lifetime SAs. DERS remained a significant predictor when controlling for homelessness, deployment, sexual harassment, and postdeployment social support, but not when controlling for depression and PTSD symptoms.	5.5
Denning et al., 2022 [34]	USA	201 patients with eating disorders in a partial hospitalization program	Cross-sectional	Suicide risk and lifetime SA through MINI-suicidality module. The total score was used to classify participants into three clinical risk categories: low, moderate, and high risk.	At a bivariate level, all DERS subscales, except for “Awareness”, were significantly associated with suicide risk severity and frequency of lifetime SAs. Limited access to adaptive ER strategies, difficulties engaging in goal-oriented behaviors, and engaging in impulsive behavior when experiencing negative emotions were associated with SA frequency. ED was not associated with suicide risk severity, even when controlling for depressive symptoms.	4
Dixon-Gordon et al., 2014 [35]	USA	246 patients admitted to a SUD residential treatment facility	Cross-sectional	SA through LPC	There was no significant relationship between DERS and SA history.	4
Duggan et al., 2013 [36]	Canada	202 university students (101 reporting lifetime NSSI + 101 matched controls)	Cross-sectional	SI related to bodily experiences through BIAI	BIAI suicide-related rumination was significantly associated with DERS scores. DERS scores significantly mediated the relationship between suicide-related rumination and NSSI.	4
Fadoir et al., 2019 [37]	USA	228 offenders	Cross-sectional	SI and SA through items 2 and 4 of SBQ-R	There were weak but significant positive correlations between DERS and SI. DERS, rumination, recent ideation, and NSSI mediated the relationship between psychopathy and SI.	3
Ghorbani et al., 2017 [38]	Iran	205 treatment-seeking alcohol dependent outpatients + 100 healthy controls	Cross-sectional	Suicide risk through the SSI. SA through anamnestic questions	Patients with lifetime SA showed higher scores on DERS. Suicide risk showed significant and positive correlations with all of the subscales of DERS. The results of the binary logistic regression showed that two dimensions of DERS (impulsivity and difficulties in goal-directed behaviors) were significant predictors of suicide risk.	4.5
Gomez-Exposito et al., 2016 [39]	Spain	122 female patients from an eating disorder unit	Cross-sectional	SA through a dichotomic question	DERS scores were significantly higher in those with a history of SA.	2
Haliczer et al., 2021 [40]	USA	181 undergraduate students	Cross-sectional	Suicide risk (including lifetime SI and SA) through SBQ-R	The total score of DERS was significantly and positively associated with suicide risk. The findings revealed an interaction between emotion dysregulation and self-damaging behaviors for suicide risk, with engagement in more forms of self-damaging behaviors (NSSI, eating disorders, alcohol, and drug use) conferring a higher risk for suicide, particularly in the context of greater emotion dysregulation. Moreover, greater emotion dysregulation had an indirect effect on elevated suicide risk via the number of self-damaging behaviors.	2.5
Harris et al., 2018 [41]	USA	1046 psychiatric outpatients (160 with previous SA)	Cross-sectional	SA through a dichotomic question	Significantly higher total DERS scores were found in patients with previous SAs. DERS was not independently associated with SA history.	3
Khazem and Anestis, 2016 [42]	USA	378 adults recruited online	Cross-sectional	SI and SA through 2 items of SITBI	Those with SI or SA had significantly higher mean DERS scores than participants without SI or SA.	4
Lemaigre and Taylor, 2019 [43]	Scotland	86 males from a community group for past or current suicidality	Cross-sectional	SI and SA through SBQ-R	There was a significant positive correlation between DERS and both SI and SA. DERS total score and social inhibition mediated the relationship between CT and SA.	4.5
Mallorquì-Bagué et al., 2018 [44]	Spain	249 inpatients with GD	Cross-sectional	SI and SA through clinical interview	DERS scores were significantly higher in the SI group. There was no significant difference in DERS scores in this group between those with SA and those without SA. DERS and GD severity indirectly increased the risk of SI through psychopathological symptoms.	4
Martin et al., 2017 [45]	USA	128 psychiatric inpatients	Cross-sectional	SA, suicidality at admission and suicidality during hospitalization through BSS	There were significant positive correlations between DERS, suicidality upon admission, and SI severity (not significant when controlling for other variables). No significant correlation between DERS and SA, or suicidality during hospitalization.	3
Mata-Greve et al., 2022 [46]	USA	387 LGBTQ (203) and/or BIPOC (246) individuals	Longitudinal	SB through SBQ-R	ED measured at T2 was positively related to SB at T3 in both populations. In both samples, the total indirect effect of cultural stressors of suicide at T1 on SB at T3 via DERS at T2 was not significant.	4.5
Miranda et al., 2013 [47]	USA	143 university students	Longitudinal	SI through BSS SA through a dichotomic question	There was a significant difference in DERS scores between those with multiple SAs, one SA, and no SA. There was a weak but significant positive correlation between baseline DERS and BSS at follow-up and a moderate positive correlation between baseline DERS and baseline SA history.	4
Mohammadzadeh et al., 2019 [48]	Iran	310 males with SUD	Cross-sectional	SI through SSI Lifetime SA through anamnestic questions	All DERS dimensions were significantly associated with SI. CT was indirectly associated with suicidal ideation through some DERS dimensions (Nonacceptance, Strategies, and Awareness).	3.5
Neacsiu et al., 2018 [49]	USA	Study 1: 120 adults Study 2: 95 adults (51 with MDD + 44 healthy controls)	Cross-sectional	Study 1: SI through ASIQ Study 2: SA through SASII	In study 1, DERS was a significant predictor of SI and accounted for 11% of the variance. In study 2, those with a SA history had significantly higher DERS scores.	4.5
Orr et al., 2020 [50]	USA	431 opioid-using adults with chronic pain	Cross-sectional	SI through item 9 of BDI	ED was significantly and positively correlated with SI. The results indicated a significant indirect relationship between cannabis-use problems and SI through ED.	3.5
Palagini et al., 2019 [51]	Italy	77 patients with BD-II	Cross-sectional	SI through SSI	There was a significant positive correlation between DERS and SI.	3.5
Palagini et al., 2019 [52]	Italy	85 patients with BD-II + 35 healthy controls	Cross-sectional	SI through SSI	There was no correlation between suicidality and DERS in the group of healthy controls. In those with BD, there was a significant positive correlation between suicidality and DERS. DERS mediated the association between disrhythmicity of social aspects and suicidality.	4.5
Palagini et al., 2022 [53]	Italy	197 psychiatric inpatients with BD	Cross-sectional	SI through SSI	SI showed significant correlations with DERS total score and Impulsivity, Awareness, and Strategies dimensions. According to linear regression analyses, passive SI was related to several DERS dimensions (Strategies and Impulsivity). The dimension of Strategies was a significant predictor of active SI and suicide plans. The results of the mediation analyses showed that the DERS total score acted as a mediator between chronobiological disrythmicity and suicidal risk. Finally, the DERS total score and the dimension of Impulsivity acted as mediators between depressive symptoms and suicidal risk.	2.5
Pisetsky et al., 2017 [54]	USA	110 patients in treatment for eating disorders	Cross-sectional	SA through 1 dichotomic question adapted from the SITBI	None of the DERS scores significantly differed between those with and without a lifetime history of SA.	2.5
Ponzoni et al., 2021 [55]	Italy	70 individuals with SI + 154 community participants	Cross-sectional	SI through BSS	Significant positive correlation between SI, DERS total score, and all DERS dimensions, except for Awareness. Moreover, DERS total score fully mediated the relationship between vulnerable narcissism and SI.	4
Raudales et al., 2023 [56]	USA	362 psychiatric inpatients with PTSD	Longitudinal	SI and SA through C-SSRS assessed at 6-week postdischarge	ED at the 3-week follow-up was significantly and positively associated with SI, but not SAs at the 6-week follow-up. The association between PTSD and ED was significant, as was the association between ED and SI. Furthermore, the indirect effect of PTSD on SI through ED was also significant, while the direct effect linking PTSD and SI was not significant when accounting for ED. The association between PTSD and ED was significant while the association between ED and SA was not significant. The indirect effect of PTSD on SA through ED was also not significant, nor was the direct effect linking PTSD and SA when accounting for ED. Significant indirect effects of PTSD on SI through ED were found for the dimensions “Goal”, “Impulse”, “Strategies”, and ”Clarity”, while the subscales “Accept” and ”Awareness” were not significant.	5
Rodriguez-Cano et al., 2022 [57]	USA	371 Spanish-speaking daily smokers	Cross-sectional	SI through item 9 of BDI	SI was significantly and positively correlated with the DERS total score. There was a positive and statistically significant main effect of ED on SI. Moreover, there was a statistically significant interaction of ED and hazardous drinking status; ED was significantly related to SI among hazardous drinkers.	3.5
Rogers and Joiner, 2018 [58]	USA	300 undergraduate students	Cross-sectional	SI and SA through SRS and BSS	There was a significant but weak positive correlation between DERS and previous SAs. There was a significant moderate correlation between DERS and SI. Logistic regression suggested that DERS was unrelated to lifetime SA.	3
Rogers et al., 2020 [59]	USA	167 undergraduate students who reported SI and/or SA	Cross-sectional	SI through BSS and DSI-SS SAs, method and lethality through a semistructured interview adapted from SITBI and BLS	ED total score differed between aborted, interrupted, and actual attempters, specifically those with higher mean scores.	2.5
Rufino et al., 2018 [60]	USA	432 psychiatric inpatients	Cross-sectional	SI and SA through C-SSRS	Significant positive correlations existed between DERS and passive SI, active SI, and SI intensity. In ANCOVA, the covariate DERS was significantly related to SI.	3.5
Rufino et al., 2020 [61]	USA	2683 psychiatric inpatients	Cross-sectional	Number of SA through 1 dichotomic question	ED, nightmare frequency, and their interaction were significantly related to previous SAs. This interaction was significant at moderate and high levels of ED, but not at low levels. Furthermore, the interaction of nightmare frequency and DERS subscales was significant for each dimension, except for difficulties engaging in goal-directed behavior at moderate and high levels.	2
Silvers et al., 2016 [62]	USA	60 females with BPD	Cross-sectional	SI and SA through CSHF	No significant difference between DERS scores in those with and without SA.	4
Turton et al., 2022 [63]	UK	65 adults with SI in the past year	Cross-sectional	SI and number of previous SA through BSS	There was a significant positive correlation between DERS total score and SI, while only 3 subscales of DERS (“Goal”, “Impulse”, and “Strategies”) were positively related to SI. The 2 models where ED was tested as a mediator between avoidant or anxious attachment and SI were insignificant. Finally, individuals with multiple SAs reported higher ED than those without a history of SA but there was no significant difference in ED between single and multiple attempters or between single and nonattempters.	4.5
Van Eck et al., 2015 [64]	USA	627 undergraduate students	Cross-sectional	SI through 1 item of the BSI	Students who reported SI had significantly higher scores on ED constructs. Moreover, results of moderated mediation models with the single dimensions of DERS have shown that ED moderated the indirect effect of ADHD on SI.	3
Yang et al., 2021 [65]	China	1596 undergraduate students	Cross-sectional	Passive and active SI through 2 items of BSS SA through 1 item of SITBI	Both suicide ideators and suicide attempters scored significantly higher on DERS than nonideators and suicide attempters scored significantly higher on ED than suicide ideators. Both suicide ideators and suicide attempters scored significantly higher on “Goal”, “Impulse”, and “Strategies”, suicide ideators scored significantly higher on “Clarity” than nonideators, while suicide attempters scored significantly higher on “Acceptance” and “Impulse” than suicide ideators. According to the results of the multinomial regression model, the dimensions of “Goal”, “Clarity”, and “Strategies” significantly discriminated suicide ideators from nonideators, “Impulse” significantly discriminated suicide attempters from nonideators, and “Awareness”, and “Clarity”, and “Impulse” significantly discriminated suicide attempters from suicide ideators.	3
Yang et al., 2022 [66]	China	1491 male prisoners (316 with probable ASPD and 169 with probable BPD)	Cross-sectional	SI and SA through SITBI	For the sample with ASPD, all DERS dimensions showed positive and significant correlations with lifetime and last year SI and with the previous year’s suicide plans, while only “Impulse” and ”Strategies” showed a correlation with the previous year’s SAs. For the sample with BPD, all DERS dimensions showed positive and significant correlations with the previous year SI; “Goals”, ”Impulse”, and ”Strategies” were related to lifetime SI; “Nonacceptance”, “Strategies”, and “Impulse” were related to last year suicide plans; and none of the subscales of DERS showed a significant correlation with the previous year’s SAs.	3.5
Yoon et al., 2022 [67]	Korea	150 Emergency room patients with SI (76) or SA (74)	Cross-sectional	SI and SA through anamnestic questions	There were no differences in ED between suicide ideators and suicide attempters.	3
Zeifman et al., 2020 [68]	Canada	130 university students	Cross-sectional	SI through BSS	Both DERS total score and all the subscales were significantly and positively associated with SI. Furthermore, the DERS total score indirectly affected the positive relationship between perfectionistic concerns, strivings, and SI. Two dimensions of ED (“Strategies” and “Clarity”) were the only significant indirect effects of the relationship between perfectionistic concerns and SI, while “Strategies” was the only significant indirect effect of the relationship between perfectionistic strivings and SI.	2
Zemestani et al., 2023 [69]	Iran	679 university students	Cross-sectional	SI through the first 5 items of BSS	DERS total score and all its dimension were positively and significantly associated with SI. The results of the mediation model revealed a significant direct effect of sleep disturbance on SI severity. Additionally, they supported the indirect relationship between sleep disturbance and SI severity mediated by ED.	2
Zobel et al., 2023 [70]	Italy	1202 community participants	Cross-sectional	SI and SA through BSS	DERS total score and all its dimensions were positively and significantly associated with SI. The results indicated that levels of ED partially mediated the pathways by which SI leads to NSSI.	3

Legend: ADHD = Attention Deficit and Hyperactivity Disorder; ASIQ = Adult Suicide Ideation Questionnaire; ASPD = Antisocial Personality Disorder; BD = bipolar disorder; BIAI = Body Influence Assessment Inventory; BIPOC = black, indigenous and people of color; BLS = Beck Lethality Scale; BPD = Borderline Personality Disorder; BSI = Brief Symptoms Inventory; BSS = Beck Scale for Suicide Ideation; CSHF = Columbia Suicide History Form; C-SSRS = Columbia-Suicide Severity Rating Scale; CT = childhood trauma; DERS = Difficulties in Emotional Regulation; DSI-SS = Depressive Symptoms Inventory—Suicidality Subscale; ED = emotional dysregulation; ER = emotional regulation; GD = gambling disorder; L-SASI = Lifetime Suicide Attempt Self Injury Interview; LGBTQ = lesbian, gay, bisexual, transgender and queer; LPC = Lifetime Parasuicide Count; MDD = major depressive disorder; MEPOS = Measure of Episodic Planning of Suicide to assess attempts; NSSI = nonsuicidal self-injuries; PHQ-9 = Patient Health Questionnaire; SA = suicide attempt; SASII = Suicide Attempt Self-Injury Interview; SB = suicide behaviors; SBQ-14 = Suicidal Behavior Questionnaire; SBQ-R = Suicidal Behavior Questionnaire-Revised; SI = suicidal ideation; SITBI = Self-Injurious Thoughts and Behaviors Interview; SRS = Suicide Rumination Scale; SSI = Scale of Suicide Ideation; SUD = substance-use disorder.

## Data Availability

Not applicable.
